# Emotional Education in Early Onset Schizophrenia and Asperger’s Syndrome

**DOI:** 10.3390/bs10090131

**Published:** 2020-08-29

**Authors:** Alessandro Frolli, Maria Carla Ricci, Francesco Alberto Tortorelli, Antonella Cavallaro, Luana Valenzano, Angelo Rega, Francesca Felicia Operto, Giulio Corrivetti

**Affiliations:** 1 DRC—Disability Research Centre, University of International Studies of Rome, 00147 Rome, Italy; 2 FINDS—Italian Neuroscience and Developmental Disorders Foundation, 81040 Caserta, Italy; mariacarla.ricci1@gmail.com (M.C.R.); luanavalenzano@yahoo.com (L.V.); 3 SPEE—Specialization School in Cognitive Behavioral Psychotherapy for Developmental Age Disorders, 80142 Naples, Italy; francescoalbertotortorelli@gmail.com (F.A.T.); a-cavallaro@live.it (A.C.); 4 Department of Psychology, University of Naples, 80100 Naples, Italy; angelo.rega@unina.it; 5 Department of Child Neuropsychiatry, University of Salerno, 84080 Fisciano, Italy; opertofrancesca@gmail.com; 6 ASL (SA), Department of Mental Health, 84122 Salerno, Italy; corrivetti@gmail.com

**Keywords:** emotional education, ASD, Asperger’s Syndrome, early onset schizophrenia, ToM, REBT

## Abstract

In this study, we aim to verify how emotional training can improve empathy and theory of mind (ToM) in patients diagnosed with early onset schizophrenia and Asperger’s syndrome. The study design includes 100 subjects divided into two experimental groups and two control groups. The two experimental groups followed a rational emotive behavior therapy (REBT) protocol. The two control groups instead underwent cognitive behavioral psychotherapy training. Analysis of Variance (ANOVA) was applied to analyze the difference between the Asperger’s syndrome (AS) and early onset schizophrenia (EOS) groups, pre and post training. Our analysis shows that the AS group improved post emotional training but only when emotions were internalized, as demonstrated by the improvement of the scores in the post-treatment eye test (ET) but not in the emotional quotient (EQ) test. The EOS group instead showed post-training improvement, not only concerning skills leading to internalizing emotions but also in empathy, as demonstrated by the improvement of EQ and Reflective Functioning Questionnaire (RFQ) test scores. These scores remained lower than in the control group. Finally, our findings reveal that the value of the treatment was more considerable for the EOS group than for the AS group due to the improvement in first- and second-order ToM skills and an improvement of empathic skills in the first group, followed by the group comprising AS subjects. In the AS group, the treatment only favored the enhancement of first-order ToM skills; however, this improved quality of life and social adaptation.

## 1. Introduction

Autism Spectrum Disorders are characterized by a deficit in social–pragmatic communication and by restricted and repetitive behavior; these disorders are distributed within a “spectrum” of phenotypic variabilities. DSM 5 [1] also specifies the level of severity of the symptoms, classifying the subject help requirements as follows: (i) very significant, (ii) significant, or (iii) modest. Schizophrenia instead refers to a clinical profile characterized by cognitive, behavioral, and emotional dysfunctions, such as delusions and hallucinations, associated with frequent disorganized speech (DSM 5) [1]. The root cause of these pathologies presents a deficit of theory of mind (ToM).

ToM is the ability to attribute mental states such as emotions, desires, knowledge or beliefs to oneself and others. The development of the ToM helps in understanding other individuals’ behavior, attributing meaning to their actions when they are observed, even if they do not provide any direct explanation. In fact, on the one hand, human behavior is guided by knowledge of reality, and on the other hand by metacognitive monitoring exploiting recursive thoughts as a vehicle (metarepresentation of thoughts) [2]. Therefore, this process can lead to a more or less accurate explanation or prediction of behavior. A first order of ToM can be distinguished, including those whose competences include the ability to understand thoughts, desires, beliefs, and recognition of one’s own and others’ emotions, and a second order of ToM can be distinguished, including higher-order skills such as self-regulation, self-monitoring, and the abilities of metarepresentation and metacognition.

Difficulties in understanding one’s own and others’ mental states and social interactions are manifested in various pathologies; some of these comprise developmental age pathologies, such as autism and schizophrenia. According to numerous studies conducted on autism spectrum disorders [3,4], these subjects underperformed in the ability to reflect on their own and others’ mental states (mentalization deficit) [5]. Subjects affected by schizophrenia are instead individuals who, despite the symptomatology, developed adequate ToM skills but have lost their second-order metarepresentations [6,7]. Metarepresentation impairment is caused by the degeneration of a neuronal population in the orbito-frontal cortex [8,9]. In fact, some studies [10,11] prove that these brain regions are strongly involved, especially the medial orbit-frontal circuit [9], which has the function of mediating empathic and socially appropriate responses. A dysfunction in this circuit can cause impairment such as emotional lability, impulsivity, irascibility, inappropriate social responses, and lack of empathy and judgment. In addition, the individual has difficulty in understanding actions’ aims, in line with the symptoms present in schizophrenia [12,13].

For a better understanding of these mechanisms, it is necessary to investigate the higher cognitive functions at the brain level related to the frontal area, which perform crucial work regarding executive functions, social behavior, self-awareness, and other-awareness. This is confirmed by data collected by functional imaging techniques [14]. Research shows a functional relationship with frontal structures having a decisive role for the function of the theory of mind [15]. A specific clinical sub-type of schizophrenia is represented by early onset schizophrenia (EOS), which is characterized by onset no older than 18 years. This can be classified in two sub-types: prepuberal, starting before the age of 12 (childhood schizophrenia, COS), and adolescent onset schizophrenia (or AdOS), with onset between the ages of 13 and 18 [12,16]. 

The research objective was to test emotional training, based on knowing, interpreting, and recognizing emotions, on subjects with EOS and subjects with Asperger’s syndrome (AS), in order to improve first-order and second-order ToM and therefore empathy. For training purposes, a modified version was used of the Ellis rational emotive behavior training (REBT) protocol [17]. This protocol not only improves the individual’s emotional capacity but also replaces irrational thoughts, eliminating bias and cognitive distortions [18]. We assessed first-order ToM and empathy with the use of an eye test [19], while for monitoring second-order ToM we used the empathy quotient test [20] and the Reflective Functioning Questionnaire 8 [21]. All tests were performed prior to treatment and repeated in post treatment. Moreover, both EOS and AS control groups took part in a treatment efficacy assessment, and both control groups were treated with cognitive behavioral psychotherapy not based on rational emotional therapy. As part of the expected changes, we predicted an improvement in the interpersonal life of the subjects, in their self-image and image of others, with repercussions for self-esteem and sense of self-effectiveness, crucial elements in therapeutic–rehabilitation interventions in clinical settings.

## 2. Materials and Methods

### 2.1. Participants

This study involved the blind participation of 100 subjects divided into four groups of 25 subjects: two experimental and two control. Participants comprised Caucasian subjects born in Italy and residing between Naples and Salerno. The data were collected by a qualified team of psychologists at the FINDS Neuropsychiatry Clinic and at the ASL Psychopathology Clinic in Salerno. The first experimental group comprised 25 subjects (19 male and 6 female) diagnosed with Asperger’s syndrome (ASe) (DSM-IV-TR, 2000) with a mean age value of 16.50. The second experimental group comprised 25 subjects (18 male and 7 female) diagnosed with early onset schizophrenia (EOSe) (DSM-IV-TR, 2000) with a mean age value of 16.33. The third AS group (ASc) comprised 25 subjects (19 male and 6 female) with a mean age value of 16.54, and the fourth EOS group (EOSc) comprised 25 subjects (17 male and 8 female) with a mean age value of 16.33. The inclusion criteria for AS groups (ASe and ASc) were as follows: (a) AS diagnosis (DSM-IV-TR, 2000), (b) participants aged between 16 and 17, (c) IQ not less than 100, as assessed through the Wechsler Adult Intelligence Scale (WAIS-IV), and (d) absence of comorbidities or defined genetic syndromes. The inclusion criteria for EOS groups (EOSe and EOSc) were: (a) diagnosis of EOS (DSM-IV-TR, 2000), (b) participants aged between 16 and 17, (c) IQ not less than 100, as assessed through WAIS-IV, and (d) absence of comorbidities or defined genetic syndromes. The population of subjects with EOS was on drug therapy with Ariprazole (10 mg/day for 6 months); non-responders for positive symptoms and those who, despite using an atypical antipsychotic, developed negative symptoms were excluded. During the study, a quarterly follow-up was performed to verify the absence of positive and negative symptoms. The evaluation of these symptoms was carried out through the administration of the Kiddie Schedule for Affective Disorders and Schizophrenia Present and Lifetime version (K-SADS-PL) diagnostic interview, which revealed scores of 0 and T0 (time of recruitment). The evaluation of the negative and positive symptoms of schizophrenia through the K-SADS-PL scales was repeated monthly until T1 was reached, and the scores always remained at 0 (absence of clinically significant symptomatology). In case of the appearance of negative or positive symptoms, the subjects would have been excluded from the sample; drug therapy was maintained unchanged throughout the whole study (Table 1).

### 2.2. Procedures and Tasks

The protocol included the following tests: Autism Diagnostic Observation Schedule (ADOS) 2 Module 3 [22], Krug Asperger’s Disorder Index [23], Autism Diagnostic Interview-Revised (ADI-R) [24], K-SADS-PL [25], Standard Progressive Matrices (SPM) [26], the Wechsler Adult Intelligence Scale (WAIS-IV) [27], the EQ test [20], and the ET [19] and *RFQ-8 (Reflective Functioning Questionnaire)* [21]: 

*ADOS 2 (Autism Diagnostic Observation Schedule) Module 3*: a structured observation consisting of 14 activities with 28 related scores. Activities focus on the social, communicative, and linguistic behavior of young and verbally fluent subjects. The module consists of three main objectives: observing the subject’s spontaneous communication when a situation provides a stimulus to communicate or interact; evaluating the subject’s ability to behave appropriately, depending on needs arising in a particular situations (i.e., telling a story, teaching a task); and observing the subject’s sense of humor and creativity.

*KADI (Krug Asperger’s Disorder Index)*: an interview for caregivers to screen symptoms related to Asperger’s syndrome and discriminate between Asperger’s and high-functioning autism following DSM-IV criteria. KADI is composed of 32 items used to identify the presence or absence of behavior indicative of AS.

*ADI-R (Autism Diagnostic Interview-Revised)*: a semi-structured interview addressed to caregivers to evaluate the presence of possible autism or autism spectrum disorders. It is composed of 93 items, investigating current behaviors and those adopted between 4 and 5 years of age to identify restricted and repetitive behavior, anomalies in social interaction, and anomalies in communication and language.

*K-SADS (Kiddie Schedule for Affective Disorders and Schizophrenia)*: a semi-structured interview aimed at identifying affective disorders, such as depression, bipolar disorder, anxiety disorder, and schizophrenia, in school-aged children from 6 to 18. The items of this interview are based on DSM-IV criteria. Currently, there are three different versions that can be undertaken by the child, the parents, or the guardians.

*SPM (Standard Progressive Matrices)*: a non-verbal test used to measure reasoning and fluid intelligence. It is composed of 60 items ranked by difficulty level, each a visual geometric design with a missing piece such that the subject has six to eight choices to pick from to fill in the missing piece. In this specific case, through the administration of the diagnostic interview, we were able to detect an absence of positive and negative symptoms in subjects with EOS, highlighted by the scores of 0 that emerged from the aforementioned scale.

*EQ (Empathy Quotient)*: a self-report questionnaire used to measure cognitive, affective, and behavioral aspects of empathy. The questionnaire consists of 40 items that concern empathic behaviors measured on a four-point Likert scale going from “Absolutely agree” to “Absolutely disagree”. Clinically, it can be used to assess the level of social impairment in certain disorders like autism disorder. According to the authors [20], empathy is a combination of the ability to feel an appropriate emotion in response to another’s emotions and the ability to understand the other’s emotions (related to ToM).

*ET (Eye Test)*: used to assess the ability to attribute complex mental states in adults with and without severe mental deficiency; it is also an appropriate tool for clinical investigation of the integrity of ToM in patients with schizophrenia. The test consists of the usage of 37 black-and-white photographs, 36 test stimuli, and a trial stimulus: the photographs portray the ocular area of adult, young, and elderly individuals of both sexes.

*RFQ-8 (Reflective Functioning Questionnaire)*: a questionnaire on the level of mentalization possessed by young patients. It is composed of two subscales: RFQ_C, which assesses the level of certainty, and RFQ_U, which assesses uncertainty about the ability to infer the mental states of oneself and others. The detection of high scores on these two scales is the index of hypermentalization disorder and hypomentalization disorder, respectively. Hypermentalization is the tendency to attribute mental states incorrectly, without any empirical evidence, whereas hypomentalization indicates a low rate of inferences of mental states, one’s own and others’ (probably in the presence of psychopathologies).

### 2.3. Procedures

All groups, both experimental and control, undertook the Raven matrices [26] test to monitor cognitive efficiency and WAIS-IV for sample recruitment (the IQ inclusion criterion was not less than 100), revealing an IQ in the norm, between the 50th and 90th percentile. The ASe and ASc subjects were diagnosed with Asperger’s syndrome (AS); this was confirmed by a follow-up clinical assessment using the criteria of DSM-IV-TR (2000). This assessment included: ADOS 2 Module 3, KADI, ADI-R, and K-SADS-PL. The diagnosis of schizophrenia (EOS) for the groups EOSe and EOSc was assessed by a clinical interview followed by observation through K-SADS-PL. The diagnostic evaluation scale was then also used for monitoring positive and negative symptoms during the period between T0 and T1. It should be noted that at the time of recruitment, patients with EOS had already started drug therapy with Ariprazole (10 mg/day for 6 months) and no longer presented negative or positive symptoms. The absence of these symptoms was confirmed monthly through the administration of K-SADS-PL and the finding of scores equal to 0 each month. ADOS 2, KADI, and ADI-R were also used to exclude the possibility of comorbidity with autism spectrum disorder. The two experimental groups ASe and EOSe underwent emotional training. This training was carried out following Ellis’s REBT protocol [17] but using Di Pietro’s revisions [28]. The emotional training consisted of several phases: (1) recognition of emotions, (2) distinction of emotions, (3) extension of emotional vocabulary, (4) discrimination of emotional gradation, (5) physical sensory experiences for emotional implementation and emotional recognition of oneself, and (6) own emotional body representation distinct from that of others. The training was carried out either with individual or group meetings. The individual training was carried out on a weekly basis and was mainly focused on active listening, validation, and psycho-education. The group training, based on circle time and roleplay, took place every fortnight. The treatment lasted 6 months. The effectiveness of emotional training was assessed by taking measurements in two different time phases: pre-test (T0) and post-test (T1). The two groups ASc and EOSc instead underwent cognitive behavioral psychotherapy training in the absence of rational emotional behavior training (REBT). The treatment involved a weekly session for 6 months. In addition, in this case a pre- and post-test evaluation was carried out. All groups undertook the EQ (Empathy Quotient) test, the RFQ-8 test, and the ET (Eyes Test) pre and post the emotional training. In particular, ET was used to assess for first-order ToM skills (ToM I) while EQ and RFQ-8 were used to assess for second-order ToM skills (ToM II and empathy).

### 2.4. Methods

Data analysis was performed using the statistical survey software SPSS 25.0 (2017) [29] (ARMONK, NY e CHICAGO). Significance was accepted at the level of 5% (*p* < 0.05). To analyze the effectiveness of the treatment, we used the Analysis of Variance (ANOVA) test, a parametric test that allows one to examine two or more data groups by comparing the variability within the groups with the variability between the groups. This analysis normally applies distributed test variables such as Fisher’s random variable F with the aim of analyzing the difference in the AS and EOS groups pre and post training. For the purpose of the study, we grouped patients in two groups: Group 1, subjects with Asperger’s syndrome (ASe—experimental group) who performed emotional training; Group 2, subjects with schizophrenia (EOSe—experimental group) who performed REBT training; Group 3, the AS group who did not perform REBT training (ASc); and Group 4, the group of schizophrenic subjects (EOSc) who did not perform emotional training.

## 3. Results

**Figure 1:**
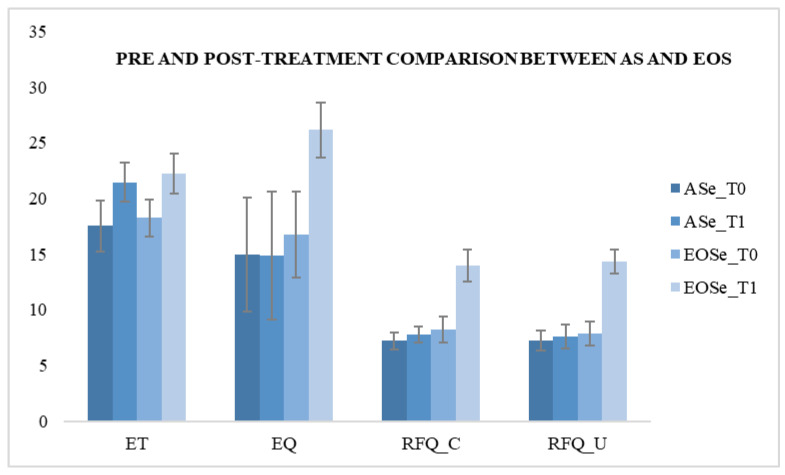
Comparison between autism spectrum group ASe and early onset schizophrenia group EOSe at T0 and T1.

For the ASe group and ASc group, we compared their scores at T0 and no significant differences emerged. We compared the scores of the EOSe group and EOSc group at T0 and no significant differences emerged (Table 2).For group ASe, we compared T0 and T1 scores and found a significant improvement only after treatment in the ET (F (1.49) = 45.107; *p* < 0.05), demonstrating an improvement of first-order ToM skills. No significant differences emerged between pre and post treatment in the EQ test and RFQ-8 test. For group EOSe, we compared the pre- and post-treatment scores and found a significant improvement in the ET (F (1.49) = 66,000; *p* < 0.05) and the EQ test (F (1.49) = 103.563; *p* < 0.05), demonstrating an improvement of both first- and second-order ToM skills after REBT treatment. We also compared the scores of the RFQ-8 test and found a significant improvement in the certainty subscale (F (1.49) = 240.813; *p* < 0.05) and the uncertainty subscale (F (1.49) = 428.446; *p* < 0.05) after the treatment. These results demonstrate an improvement of reflexive functions and therefore of the second-order and empathic functions of the ToM (Table 3 and Figure 1).

**Figure 2:**
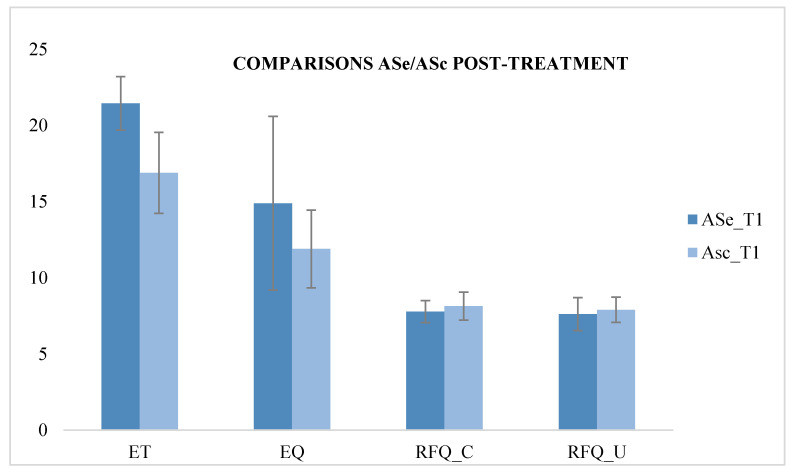
Comparison between ASe and ASc at T1.

**Figure 3:**
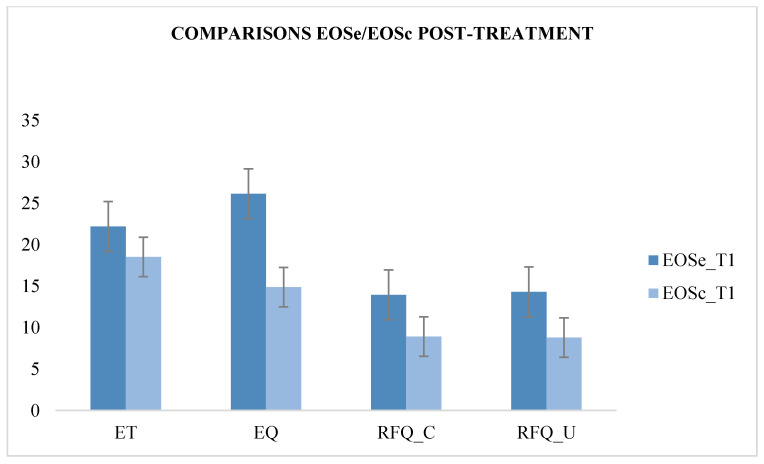
Comparison between EOSe and EOSc at T1.

We compared the scores of the ASe group with those of the ASc group at T1, and it showed a significant improvement in the ET (F (1.49) = 50.96; *p* < 0.05) in the ASe group after REBT treatment, demonstrating an improvement in first-order ToM skills compared with the ASc group’s psychotherapy treatment. We compared the scores of the EOSe group with those of the EOSc group at T1, and it showed a significant improvement in the ET (F (1.49) = 50.708; *p* < 0.05) and the EQ test (F (1.49) = 147.60; *p* < 0.05), thus demonstrating an improvement in first- and second-order ToM skills following REBT treatment in the EOSe group. Significant improvements also emerged in the RFQ test in the certainty subscale (F (1.49) = 179.73; *p* < 0.05) and in the uncertainty subscale (F (1.49) = 279.37; *p* < 0.05), demonstrating an improvement in reflexive functions after REBT treatment in the EOSe group (Table 4 and Figure 2 and Figure 3).

**Figure 4:**
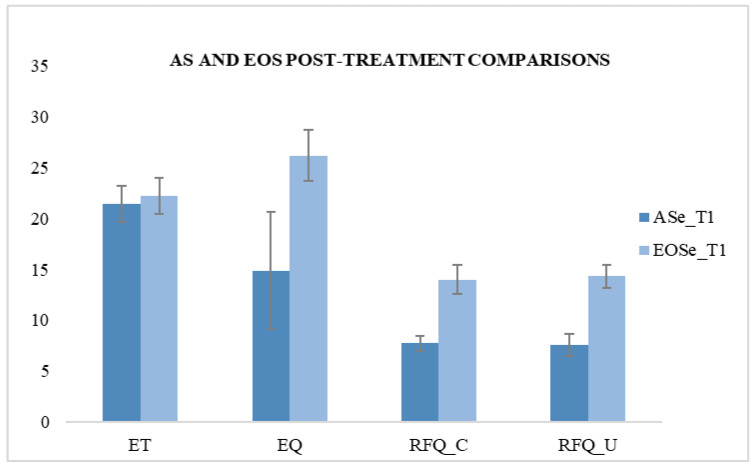
Comparison between ASe and EOSe at T1.

We then compared the T1 scores of the ASe group and the EOSe group, and it highlighted significant differences after REBT treatment, detected in the EQ test (F (1.49) = 81.738; *p* < 0.05) and the RFQ test in the certainty subscale (F (1.49) = 374.90; *p* < 0.05) and in the uncertainty subscale (F (1.49) = 471.71; *p* < 0.05) (Table 5 and Figure 4).

Finally, we compared the scores of the ASe group and the EOSe group with the scores of a normative sample on the ET and EQ test and no significant results emerged, indicating that the competences of the first- and second-order ToM remain lower than those with typical development.

## 4. Discussion

This study aimed to highlight interventions typical of evolutionary age [30] and school age [31,32], such as emotional training, in adolescent subjects who are affected by serious and disabling diseases such as EOS and AS. Difficulties in social interaction and in understanding the mental and emotional states of others, typical of schizophrenia, are similar to those experienced by subjects with autism [33,34] because in both cases there is an important metacognitive impairment, though at a different level and order. According to Baron-Cohen [4], one of the main characteristics of autism is represented by a sort of blindness to mental content; an autistic individual has deficits in perceiving the existence of mental states in other people, and therefore appears unable to provide a mental explanation of social interactions that surround and involve him. The origin of the social and communicative difficulties of autistic subjects should therefore be found in a defective maturation of this cognitive mechanism. It is possible that in autism this maturation is compromised from the beginning or that it is reached with a remarkable delay. This would explain the heterogeneity of ToM impairment and the symptomatological variability of autistic disorder, which includes both children with accentuated social impairment (low-functioning autism) and children with mild social deficits (Asperger’s syndrome) (APA) [1]. Other studies have shown that people with schizophrenia experience considerable difficulty in social interaction [21,35] and have dysfunctions in the ability to understand the ToM’s experimental tasks [36,37] similar to subjects with autism [34,38]. Particularly, in schizophrenia, ToM deficits could explain some psychotic symptoms (delirium of control, delirium of persecution, disorganization of thought, and language impairments) and behavioral symptoms (irritability, emotional dysregulation, impulsivity). Indeed, these symptoms can be better understood as a low ability of patients to relate their intentions to their own behavior and to interpret the intentions of others [2,39,40,41] explains delirium and hallucinations as an attempt to make thoughts make sense to each other, after losing the capability to represent them correctly and put them together in a cause–effect relationship.

In our analysis, we demonstrate that EOS significantly improved post emotional training compared with subjects with AS, both in the ET and in the EQ test, demonstrating the ability to improve the first- and second-order ToM skills post a specific treatment, since their difficulty was due to a partial loss of the metarepresentational ability (Ballerini) [42]. In autistic subjects, an improvement post training was evident, but they still failed to obtain an adequate performance, since they did not have the ability to develop any metarepresentational competence [33]. The scores in both cases remained lower than in the control group, demonstrating difficulty and impairment in first- and second-order ToM, even if was of a different degree in the first- and second-order ToM. The main difference between autistic and schizophrenic individuals is that the former ones show a delay in ToM acquisition [33] whereas in schizophrenia there is at first an acquisition of ToM (ToM I) but it then becomes gradually less effective [42]. Such an ability, however, is neither flexible enough nor fast or general enough to allow for an understanding of second-order metarepresentations (ToM II) and so to allow for an adequate social life and appropriate communication (APA) [1].

Frith [2] has suggested that ToM in patients with schizophrenia is compromised due to their inability to monitor their own and other’s mental states and behaviors, thus explaining many positive and negative symptoms of schizophrenia. Our analysis shows that the ASe group improved post emotional training but only in the internalization of emotions, as demonstrated by the improvement of the scores in the post-treatment ET but not in the EQ test. The EOSe group, in contrast, improved post training not only in emotional internalization skills but in empathy, as demonstrated by the improvement of EQ and RFQ-8 test scores. These scores remained lower than in the control group, indicating that an autistic individual seems to be incapable of giving a mental explanation of the social interactions that surround and involve him [4]. Our analysis also revealed an improvement in the EOSe group of reflective functions after emotional training, made evident by the significance of the RFQ-8 test scores. Conversely, in the ASe group the scores were not significant. These data indicate that rational emotional behavioral training can also help to improve the reflexive functions, second-order ToM skills, and empathy in the EOS group. In fact, both control groups that followed not REBT training but cognitive behavioral treatment demonstrated a lack of improvement in both the ET test and in the EQ and RFQ-8 tests.

Emotional training therefore represents a cognitive-behavioral rehabilitation that can improve deficient aspects such as empathic manifestation and the ability to understand other people’s mental states, with the most significant results in subjects with EOS. The reliability of the formalized measuring instruments and their employment had the objective of verifying significant qualitative results, towards empathic and relational improvement, of the training in emotional literacy of subjects suffering from serious diseases such as early onset schizophrenia and Asperger’s syndrome. This demonstrated the affordability of these instruments within different clinical contexts. The intervention of emotional literacy results in this age group is extremely important because it works on the recognition of thoughts and associated behavioral manifestations. Furthermore, it promotes the acceptance of oneself and others, a key aspect of adolescence [28]; it also lays fundamental foundations for future clinical and psychotherapeutic interventions [43,44] not only towards the awareness in qualitative and quantitative terms of the emotional world and its pathology, but to promote processes of compliance, alliance, and adherence in the clinical project [45,46].

This work not only represents a confirmation of what the literature already highlights around the symptom aspects of schizophrenia and Asperger’s syndrome, but moreover allows us to hypothesize the development of such psychotherapy techniques in the field of psychiatric rehabilitation. It is intended to sensitize the use of meaningful procedures of rehabilitation and results in the community approach to psychiatric patients at the group level. The value of the treatment is more relative to EOS than to AS, as there is an improvement of both first- and second-order ToM. The value of the treatment is more considerable for the EOS group than for the AS group due to the greater improvement in first- and second-order ToM skills and the improvement of empathic skills in the EOS group than in the AS group. In fact, in the AS group, this treatment favors a significant improvement of first-order ToM skills, which allows patients improved quality of life and better social adaptation.

## Figures and Tables

**Table 1:** Subdivision of the sample.

ASe			EOSe			ASc			EOSc		
M_age_	SD	Gerder	M_age_	SD	Gerder	M_age_	SD	Gerder	M_age_	SD	Gerder
16, 49	0, 23	M/F 19/6	16, 33	0, 46	M/F 18/7	16, 53	0, 17	M/F 19/9	16, 33	0, 46	M/F 17/8

**Table 2:** Comparison between ASe/ASc and EOSe/EOSc at T0.

	Subgroup (N = 25)								Total Sample (N = 100)							
	RFQ_C				RFQ_U				ET				EQ			
	Means	SD	F	*p*	Means	SD	F	*p*	Means	SD	F	*p*	Means	SD	F	*p*
ASe_T0	7.2	0.866			7.28	0.891			17.52	2.33			14.96	5.16		
Asc_T0	7.96	0.676			7.76	0.723			16.6	2.43			11.56	2.5		
EOSe_T0	8.24	1.16	2.982	0.091	7.88	1.09	1.887	0.176	18.24	1.64	1.597	0.212	16.76	3.88	1.93	0.170
EOSc_T0	8.4	1.08			8.12	1.09			18.12	1.81			15.68	3.54		

**Table 3:** Comparison between ASe T0/T1 (pre/post training) and EOSe T0/T1 (pre/post training).

	Subgroup (N = 25)								Total Sample (N = 100)							
	RFQ_C				RFQ_U				ET				EQ			
	Means	SD	F	*p*	Means	SD	F	*p*	Means	SD	F	*p*	Means	SD	F	*p*
ASe_T0	7.2	0.866			7.28	0.891			17.52	2.33			14.96	5.16		
ASe_T1	7.76	0.723	6.157	0.017	7.6	1.08	1.306	0.259	21.44	1.758	45.107	0.000 *	14.88	5.71	0.003	0.959
EOSe_T0	8.24	1.16			7.88	1.09			18.24	1.64			16.76	3.88		
EOSe_T1	13.96	1.42	240.813	0.000 *	14.32	1.1	428.446	0.000 *	22.2	1.8	66	0.000 *	26.16	2.49	103.563	0.000 *

* p < 0.05.

**Table 4:** Comparison between ASe/ASc at T1 and EOSe/EOSc at T1.

	Subgroup (N = 25)								Total Sample (N = 100)							
	RFQ_C				RFQ_U				ET				EQ			
	Means	SD	F	*p*	Means	SD	F	*p*	Means	SD	F	*p*	Means	SD	F	*p*
ASe_T1	7.76	0.723	2.342	0.132	7.6	1.08	1.054	0.310	21.44	1.758	50.965	0.000 *	14.88	5.718	5.737	0.021
Asc_T1	8.12	0.927			7.88	0.833			16.88	2.666			11.88	2.555		
EOSe_T1	13.96	1.428	179.728	0.000 *	14.32	1.108	279.374	0.000 *	22.2	1.803	50.708	0.000 *	26.16	2.495	147.60	0.000 *
EOSc_T1	8.92	1.222			8.8	1.225			18.52	1.851			16.04	3.335		

* p < 0.05.

**Table 5:** Comparison between ASe and EOSe at T1.

	Subgroup (N = 25)								Total Sample (N = 100)							
	RFQ_C				RFQ_U				ET				EQ			
	Means	SD	F	*p*	Means	SD	F	*p*	Means	SD	F	*p*	Means	SD	F	*p*
ASe_T1	7.76	0.723			7.6	1.08			21.44	1.758			14.88	5.718		
EOSe_T1	13.96	1.428	374.902	0.000 *	14.32	1.108	471.71	0.000 *	22.2	1.803	2.278	0.138	26.16	2.495	81.738	0.000 *

* p < 0.05.
